# Supplementary material for: Simple methods to acutely measure multiple timing metrics among the sexual repertoire of male Drosophila
Source: eLife. 2026 Nov 6;15:RP109742. doi: 10.7554/eLife.109742 (PMC13743079; doi:10.7554/eLife.109742)
Supplement: Peer review history

# Editor report: eLife Assessment

**DOI:** 10.7554/eLife.109742.3.sa0

**Authors:** Mani Ramaswami

**Affiliations:** Trinity College Dublin Ireland

**Keywords:** Convincing, Useful

This **useful** paper describes a software tool, ‘DrosoMating’, which allows automated, high-throughput quantification of six common metrics of courtship and mating behaviors in *Drosophila melanogaster*. The validity of the tool is **convincingly** shown to perform as well as expert human assessments across a wide range of conditions. While it is not suitable yet for pose-based classification of individual actions, and its utility with respect to new deep learning-based tools such as DeepLabCut and SLEAP has yet to be assessed, the efficient coarse-grained detection of behavioral states in low-resolution videos offered by DrosoMating represents a very helpful addition to analytical tools available for assessing courtship behavior in *Drosophila*.

# Referee report: Reviewer #1 (Public review)

**DOI:** 10.7554/eLife.109742.3.sa1

Summary:

The study of Drosophila mating behaviors has offered a powerful entry point for understanding how complex innate behaviors are instantiated in the brain. The effectiveness of this behavioral model stems from how readily quantifiable many components of the courtship ritual are, facilitating the fine-scale correlations between the behaviors and the circuits that underpin their implementation. Detailed quantification, however, can be both time consuming and error prone, particularly when scored manually. Song et al. have sought to address this challenge by developing DrosoMating, software that facilitates the automated and high-throughput quantification of 6 common metrics of courtship and mating behaviors. Compared to a human observer, DrosoMating matches courtship scoring with high fidelity. Further, the authors demonstrate that the software effectively detects previously described variations in courtship resulting from genetic background or social conditioning. Finally, they validate its utility in assaying the consequences of neural manipulations by silencing Kenyon cells involved in memory formation in the context of courtship conditioning.

Strengths:

(1) The authors demonstrate that for three key courtship/mating metrics, DrosoMating performs virtually indistinguishably from a human observer, with differences consistently within 10 seconds and no statistically significant differences detected. This demonstrates the software's usefulness as a tool for reducing bias and scoring time for analyses involving these metrics.

(2) The authors validate the tool across multiple genetic backgrounds and experimental manipulations to confirm its ability to detect known influences on male mating behavior.

(3) The authors present a simple, modular chamber design that is integrated with DrosoMating and allows for high throughput experimentation, capable of simultaneously analyzing up to 144 fly pairs across all chambers.

Weaknesses:

(1) DrosoMating appears to be an effective tool for the quantification of key courtship and mating metrics, but similar tools for automated analysis already exist. The authors present a compelling use case for DrosoMating, where it has particular advantages over tools like FlyTracker and Ctrax for high-throughput analysis. This comparative analysis, however, leaves out modern pose-estimation methods (SLEAP, DeepLabCut), better able to tolerate low contrast and occlusion. It therefore remains unclear what specific advantages it might offer over current machine learning approaches.

(2) The courtship behaviors of Drosophila males represent a series of complex behaviors that unfold dynamically in response to female signals. While metrics like courtship latency, courtship index, and mating duration are useful, they compress the complexity of actions that occur throughout the mating ritual. The authors suggest DrosoMating's modular architecture facilitates integration with behavioral classifiers like JAABA. Such integration could substantially expand the utility of this tool for the broader Drosophila neuroscience community, but in its current form its applications are confined to summary timing metrics.

(3) Validation is limited to multiple *D. melanogaster* strains. Cross-species studies of mating behavior diversity are increasingly common, so demonstrating the tool's accuracy across species would strengthen claims about its broader applicability.

# Referee report: Reviewer #2 (Public review)

**DOI:** 10.7554/eLife.109742.3.sa2

This manuscript introduces DrosoMating, an integrated hardware-software pipeline designed to automate quantification of Drosophila courtship and mating behavior. The authors aim to provide a low-cost, scalable alternative to existing behavioral tracking systems, focusing on extracting key temporal metrics including courtship index, copulation latency, and mating duration from high-throughput video recordings.

A major strength of the work is the clear emphasis on experimental scalability and practical usability. The system is designed for multi-chamber recording and performs robustly under low-quality imaging conditions where conventional pose-tracking pipelines often fail. The revised manuscript substantially improves its scientific positioning through the inclusion of systematic benchmarking against widely used tools (Ctrax and FlyTracker), demonstrating that both fail to complete end-to-end analysis under these recording conditions: Ctrax due to segmentation instability and trajectory fragmentation, FlyTracker due to runtime errors during feature computation. A comparative table (Table 1) summarizes key features across tools, and the authors appropriately qualify that these limitations are specific to the low-quality video conditions tested and should not be interpreted as general shortcomings of those tools. The addition of individual-level behavioral ethograms (Figure S3) further strengthens the evidence by allowing direct assessment of the system's temporal resolution at the single-fly level.

The authors also appropriately address statistical concerns raised in review. The re-analysis using ANOVA frameworks - one-way ANOVA with Tukey's HSD for multi-strain comparisons, two-way ANOVA with Sidak's correction for genotype × training interactions - improves the rigor of the behavioral comparisons and supports the revised conclusions regarding strain and learning effects. The addition of locomotor control analyses (Figure S4) further clarifies interpretation of mutant phenotypes by partially disentangling motor from courtship-specific effects, with the revised text appropriately acknowledging contributions from both general hypoactivity and sensory impairments.

A key limitation remains the conceptual scope of the system. DrosoMating is optimized for state-based temporal segmentation rather than fine-grained behavioral annotation or posture-level decomposition. While the authors now clearly acknowledge this and position the tool appropriately, it inherently restricts its use cases compared to modern pose-estimation and classifier-based frameworks. Additionally, the benchmarking comparison is necessarily asymmetric: DrosoMating is tested on low-quality videos where it excels by design, while Ctrax and FlyTracker are evaluated under conditions outside their intended operating range. A comparison under more favorable conditions for the tracking-based tools, or an evaluation of whether modest improvements in video quality would bring conventional pipelines within functional range, would further contextualize the practical boundary between approaches. The comparison also does not include modern deep-learning-based tools (e.g., DeepLabCut, SLEAP), which may be more robust to low-contrast conditions than classical segmentation-based pipelines. These points do not diminish the demonstrated utility of DrosoMating for its intended niche but would help users make more informed decisions about tool selection.

Overall, the revised manuscript presents a well-validated and clearly positioned contribution. It defines the niche in which DrosoMating provides substantial practical value while appropriately delimiting its limitations relative to more general behavioral analysis frameworks.

# Author comment: Author response

**DOI:** 10.7554/eLife.109742.3.sa3

**Authors:** Yutong Song, Hongyu Miao, Dongyu Sun, Xiao Liu, Fan Jiang, Xuejiao Yang, Woo Jae Kim

**Affiliations:** Harbin Institute of Technology Harbin China; Harbin Institute of Technology Harbin China; Harbin Institute of Technology Harbin China; Harbin Institute of Technology Harbin China; University of Science and Technology of China Hefei China; Harbin Institute of Technology Harbin China; Harbin Institute of Technology Harbin Republic of Korea

The following is the authors’ response to the original reviews.

**Public Reviews:**

**Reviewer #1 (Public review):**

Summary:

The study of Drosophila mating behaviors has offered a powerful entry point for understanding how complex innate behaviors are instantiated in the brain. The effectiveness of this behavioral model stems from how readily quantifiable many components of the courtship ritual are, facilitating the fine-scale correlations between the behaviors and the circuits that underpin their implementation. Detailed quantification, however, can be both time-consuming and error-prone, particularly when scored manually. Song et al. have sought to address this challenge by developing DrosoMating, software that facilitates the automated and high-throughput quantification of 6 common metrics of courtship and mating behaviors. Compared to a human observer, DrosoMating matches courtship scoring with high fidelity. Further, the authors demonstrate that the software effectively detects previously described variations in courtship resulting from genetic background or social conditioning. Finally, they validate its utility in assaying the consequences of neural manipulations by silencing Kenyon cells involved in memory formation in the context of courtship conditioning.

Strengths:

(1) The authors demonstrate that for three key courtship/mating metrics, DrosoMating performs virtually indistinguishably from a human observer, with differences consistently within 10 seconds and no statistically significant differences detected. This demonstrates the software's usefulness as a tool for reducing bias and scoring time for analyses involving these metrics.

(2) The authors validate the tool across multiple genetic backgrounds and experimental manipulations to confirm its ability to detect known influences on male mating behavior.

(3) The authors present a simple, modular chamber design that is integrated with DrosoMating and allows for high-throughput experimentation, capable of simultaneously analyzing up to 144 fly pairs across all chambers.

Weaknesses:

(1) DrosoMating appears to be an effective tool for the high-throughput quantification of key courtship and mating metrics, but a number of similar tools for automated analysis already exist. FlyTracker (CalTech), for instance, is a widely used software that offers a similar machine vision approach to quantifying a variety of courtship metrics. It would be valuable to understand how DrosoMating compares to such approaches and what specific advantages it might offer in terms of accuracy, ease of use, and sensitivity to experimental conditions.

(2) The courtship behaviors of Drosophila males represent a series of complex behaviors that unfold dynamically in response to female signals (Coen et al., 2014; Ning et al., 2022; Roemschied et al., 2023). While metrics like courtship latency, courtship index, and copulation duration are useful summary statistics, they compress the complexity of actions that occur throughout the mating ritual. The manuscript would be strengthened by a discussion of the potential for DrosoMating to capture more of the moment-to-moment behaviors that constitute courtship. Even without modifying the software, it would be useful to see how the data can be used in combination with machine learning classifiers like JAABA to better segment the behavioral composition of courtship and mating across genotypes and experimental manipulations. Such integration could substantially expand the utility of this tool for the broader Drosophila neuroscience community.

(3) While testing the software's capacity to function across strains is useful, it does not address the "universality" of this method. Cross-species studies of mating behavior diversity are becoming increasingly common, and it would be beneficial to know if this tool can maintain its accuracy in Drosophila species with a greater range of morphological and behavioral variation. Demonstrating the software's performance across species would strengthen claims about its broader applicability.

**Reviewer #2 (Public review):**

This paper introduces "DrosoMating," an integrated hardware and software solution for automating the analysis of male Drosophila courtship. The authors aim to provide a low-cost, accessible alternative to expensive ethological rigs by utilizing a custom acrylic chamber and smartphone-based recording. The system focuses on quantifying key temporal metrics-Courtship Index (CI), Copulation Latency (CL), and Mating Duration (MD)-and is applied to behavioral paradigms involving memory mutants (orb2, rut).

The development of open-source behavioral tools is a significant contribution to neuroethology, and the authors successfully demonstrate a system that simplifies the setup for large-scale screens. A major strength of the work is the specific focus on automating Copulation Latency and Mating Duration, metrics that are often labor-intensive to score manually.

However, there are several limitations in the current analysis and validation that affect the strength of the conclusions:

First, the statistical rigor requires substantial improvement. The analysis of multi-group experiments (e.g., comparing four distinct strains or factorial designs with genotype and training) currently relies on multiple independent Student's t-tests. This approach is statistically invalid for these experimental designs as it inflates the family-wise Type I error rate. To support the claims of strain-specific differences or learning deficits, the data must be analyzed using Analysis of Variance (ANOVA) to properly account for multiple comparisons and to explicitly test for interaction effects between genotype and training conditions.

Second, the biological validation using $w^{1118}$ and $y^1$ mutants entails a potential confound. The authors attribute the low Courtship Index in these strains to courtship-specific deficits. However, both strains are known to exhibit general locomotor sluggishness (due to visual or pigmentation/behavioral defects). Since "following" behavior is likely a component of the Courtship Index, a reduction in this metric could reflect a general motor deficit rather than a specific lack of reproductive motivation. Without controlling for general locomotion, the interpretation of these behavioral phenotypes remains ambiguous.

Third, the benchmarking of the system is currently limited to comparisons against manual scoring. Given that the field has largely adopted sophisticated open-source tracking tools (e.g., Ctrax, FlyTracker, JAABA), the utility of DrosoMating would be better contextualized by comparing its performance - in terms of accuracy, speed, or identity maintenance - against these existing automated standards, rather than solely against human observation.

Finally, the visual presentation of the data hinders the assessment of the system's temporal precision. While the system is designed to capture time-resolved metrics, the results are presented primarily as aggregate bar plots. The absence of behavioral ethograms or raster plots makes it difficult to verify the software's ability to accurately detect specific transitions, such as the exact onset of copulation.

We sincerely thank the reviewers for their constructive and detailed feedback, which has substantially improved the clarity and rigor of our work. Below is a summary of the major revisions.

(1) Comparison with existing tools. We added a new main figure (Figure 5) and Table 1 systematically benchmarking DrosoMating against Ctrax and FlyTracker on identical low-quality, high-throughput mating videos. Both conventional tools failed under our recording conditions: Ctrax showed severe segmentation instability and fragmented trajectories, while FlyTracker frequently crashed during feature computation. These results demonstrate that DrosoMating's state-detection approach bypasses the pose-tracking limitations that impair established pipelines. We also revised the Discussion to clarify that DrosoMating is specialized for robust extraction of mating timing metrics from low-quality videos, not a replacement for general-purpose tracking tools.

(2) Statistical analysis. Following Reviewer #2's recommendation, we re-analyzed Figure 3 using one-way ANOVA with Tukey's HSD for strain comparisons, and Figure 4 using two-way ANOVA with Sidak's post-hoc test for learning assays, including the critical Genotype by Training interaction term.

(3) New supplementary data. We added Figure S4 showing basal locomotor velocity of single-housed males across all four strains to decouple motor defects from courtship deficits in w1118 and y1 mutants. We also added Figure S3 with behavioral ethograms for individual flies to visually demonstrate the system's temporal resolution and detection accuracy.

(4) Additional improvements. We standardized statistical reporting across all figure legends, explicitly defined the segmentation threshold parameter s in the Methods, consistently used "Mating Duration (MD)" throughout, standardized MB247-GAL4 labeling, clarified that occluded frames are retained for mating-state detection via merged-contour analysis, and corrected minor errors including duplicate references and unnecessary quotation marks.

We believe these revisions substantially strengthen the manuscript and clearly position DrosoMating within the existing ecosystem of behavioral analysis tools.

We remain grateful for the valuable feedback from both reviewers and the editorial team, and we hope the revised version meets the standards for publication in eLife.

**Recommendations for the authors:**

**Reviewer #1 (Recommendations for the authors):**

(1) It's difficult to assess the utility of this tool in relation to the variety of alternative methods available for the automated scoring of courtship behavior, some of which offer more granular behavioral data than what DrosoMating has been presented to produce. A direct comparison to other approaches (e.g., FlyTracker, DeepLabCut, SLEAP) would be highly valuable for understanding what use cases DrosoMating is best suited for. Ideally, this analysis would include (i) a comparison of the accuracy of scoring courtship metrics and constituent behaviors, (ii) an assessment of the sensitivity of the various software to different experimental conditions, such as lighting and alternative chamber designs, and (iii) testing across Drosophila species, which could help highlight the particular strengths of DrosoMating over more established methods. At a minimum, a table comparing key features, requirements, and capabilities would help position DrosoMating within the existing ecosystem of tools.

We sincerely appreciate the reviewer’s insightful comments on benchmarking DrosoMating against existing tools for automated courtship behavior analysis. We fully agree that direct comparisons are essential to clarify the unique advantages and ideal use cases of DrosoMating. We have extensively revised the manuscript by adding comparative experiments, a new figure (Figure 5) and comparative table (Table 1), and expanded descriptions, as detailed below.

(1) Direct comparison with conventional tracking tools

We systematically tested Ctrax and FlyTracker on the same low-quality, high-throughput mating videos used for DrosoMating. The corresponding results are presented in Figure 5.

Ctrax showed severe segmentation instability, including over-segmentation, under-segmentation, and fragmented trajectories, and failed to stably detect two flies.

FlyTracker was able to generate background models and showed partially improved segmentation after threshold adjustment, but frequently crashed during feature computation and could not complete the end-to-end analysis pipeline.

These results confirm that tracking-based tools are vulnerable to low contrast, chamber artifacts, and prolonged male–female overlap, whereas DrosoMating bypasses pose tracking and directly detects mating states.

The revised manuscript text is as follows (line 265-342):

"DrosoMating is more compatible with low-quality mating videos than conventional tracking-based pipelines.

To evaluate whether conventional fly-tracking pipelines could be used as alternative tools for extracting mating-duration metrics, we tested Ctrax and FlyTracker on representative low-quality single-chamber mating videos recorded under our standard high-throughput conditions (Fig. 5). Ctrax is designed to estimate the position and orientation of multiple walking flies while maintaining individual identities over time (Branson et al., 2009) (https://ctrax.sourceforge.net/), whereas FlyTracker aims to track fly pose, including position, orientation, body size, wing and leg positions, and to generate trajectory- and feature-based outputs for downstream behavior analysis (Eyjolfsdottir et al., 2014). Because these programs were not readily compatible with our full high-throughput behavioral recording setup, we first cropped the original videos and tested single-chamber videos containing one male and one female fly.

Using Ctrax, we observed that target detection was highly variable even within single-chamber videos. In representative frames, Ctrax could occasionally identify the two flies correctly (Fig. 5A). However, imperfect segmentation was frequently observed. In some frames, parts of the fly body were detected as additional targets, resulting in over-segmentation and an apparent increase in the number of detected flies (Fig. 5B, C). Conversely, when the male and female were close to each other or physically overlapped, the two animals were sometimes detected as a single target (Fig. 5D). These examples indicate that Ctrax detection was sensitive to the low contrast and overlapping fly bodies present in our mating videos. We then adjusted the Ctrax detection threshold to improve segmentation quality. Although threshold optimization improved detection in some frames, abnormal detections remained evident across randomly sampled frames (Fig. 5E). For example, some frames still showed incorrect target numbers, and a severe segmentation failure was observed in the lower-right example, where the detected objects did not correspond to two clearly separable flies. Thus, even after parameter optimization, Ctrax did not consistently maintain the expected two-target detection state in single-chamber mating videos.

The instability of Ctrax detection was also reflected in the trajectory output. In the first 500 frames, the generated trajectories were fragmented into multiple colored track segments rather than two continuous trajectories corresponding to the male and female (Fig. 5F). In addition, some trajectories extended outside the chamber boundary, indicating tracking errors and identity instability. Consistently, frame-by-frame quantification of detected target number showed that the detected object count did not remain stable at the expected value of two flies per chamber (Fig. 5G). Because Ctrax failed to maintain stable two-fly detection and continuous trajectories under these video conditions, its output could not be reliably used for downstream extraction of copulation latency or mating duration.

We next tested FlyTracker on the same type of cropped single-chamber mating videos. FlyTracker was developed to track multiple flies by estimating body position, orientation, size, wing and leg positions, and by maintaining fly identities across video frames; it also outputs per-frame features such as velocity, facing angle, and wing-angle-related measurements for downstream behavioral analysis (Eyjolfsdottir et al., 2014). In our videos, FlyTracker was able to generate a background model, indicating that the program could recognize the overall imaging field and chamber background (Fig. 5H). However, during calibration and segmentation, the default threshold setting produced inconsistent detection results (Fig. 5I). Although some frames were segmented relatively well under the default threshold (Fig. 5J), these successful examples were not representative of the overall tracking process, and the program frequently terminated with runtime errors during tracking or downstream feature computation. To improve detection stability, we lowered the segmentation threshold. Under this adjusted setting, FlyTracker produced more complete fly masks in representative frames (Fig. 5K), and the diagnostic output showed that two flies were detected in many sampled frames (Fig. 5L). Nevertheless, detection remained unstable in some sampled frames, including frames in which zero or one fly was detected despite the expected two flies per chamber (Fig. 5L). The full pipeline ultimately failed during feature computation, producing a runtime error before complete tracking and feature outputs could be generated (Fig. 5M). Therefore, even after threshold adjustment, FlyTracker could not provide a stable end-to-end workflow for extracting mating-duration metrics from these low-quality mating videos.

This failure mode is relevant because FlyTracker depends on stable segmentation, identity maintenance, and per-frame feature extraction. In our assay videos, low contrast, chamber-edge artifacts, and prolonged male–female overlap during copulation interfered with these requirements. As a result, FlyTracker could occasionally identify the flies in individual frames, but it did not reliably complete the full analysis pipeline required for downstream behavioral quantification. This limitation is especially important for workflows such as JAABA, which use manually labeled examples to train behavior classifiers but still depend on upstream tracking-derived features. Thus, for our low-quality high-throughput mating recordings, FlyTracker-based analysis was substantially less practical than DrosoMating, which directly outputs mating-related timing metrics without requiring continuous high-fidelity two-fly pose tracking."

(2) Evaluation of accuracy, robustness, and cross-species potential

We addressed the three key points requested by the reviewer:

(i) Accuracy: DrosoMating achieved 98–99% agreement with manual scoring. Under our experimental conditions, neither Ctrax nor FlyTracker completed an end-to-end workflow capable of reliably extracting copulation latency and mating duration. Ctrax produced fragmented trajectories with unstable target counts, and FlyTracker terminated with runtime errors during feature computation.

(ii) Robustness: DrosoMating is highly robust to low-contrast lighting and common behavioral chamber setups, whereas conventional tools require high-quality videos and fail under fly occlusion.

(iii) Cross-species testing: We did not perform cross-species validation in this revision. DrosoMating relies on mating state detection rather than species-specific morphology, suggesting potential transferability, although this remains to be experimentally validated. We have therefore revised the text to avoid claiming demonstrated cross-species performance and now describe this as a potential future application.

The revised manuscript text is as follows (line 572-576):

"Cross-species testing was not performed in this study. Although DrosoMating's state-detection approach is morphology-agnostic and therefore theoretically applicable across Drosophila species, we have revised the text to avoid claiming demonstrated cross-species performance. Formal validation across diverse species remains a promising future direction."

We added Table1 to compare key features across DrosoMating, Ctrax, and FlyTracker, including low-quality video compatibility, multi-chamber support, robustness to fly overlap, direct output of mating timing metrics, accuracy.

(3) Clarification of ideal use cases

We revised the Discussion to emphasize that DrosoMating is not intended to replace general-purpose pose or tracking tools (e.g., FlyTracker, Ctrax) that provide fine-grained behavioral data.

Instead, it offers a specialized, robust, and high-throughput workflow for scenarios requiring efficient extraction of mating timing metrics from low-quality videos, where conventional pipelines often fail.

These revisions greatly improve the clarity and positioning of DrosoMating. We thank the reviewer for this valuable suggestion.

The revised manuscript text is as follows (line 578-620):

“Comparative Evaluation with Existing Courtship Analysis Tools

A major advantage of DrosoMating is its compatibility with low-quality, high-throughput mating videos. Conventional tracking-based tools such as Ctrax and FlyTracker are powerful for trajectory- and pose-based behavioral analysis, but they generally require stable object segmentation, identity maintenance, and reliable feature extraction across frames. Ctrax was designed to estimate the positions and orientations of multiple walking flies while maintaining their identities, whereas FlyTracker aims to track detailed fly pose and generate per-frame behavioral features. Under our recording conditions, these requirements were difficult to satisfy because the videos were low contrast and the male and female frequently overlapped during copulation.

In our tests, both tools showed limited compatibility with these videos. Even after cropping to single-chamber videos and adjusting detection parameters, Ctrax produced unstable target numbers, fragmented trajectories, and tracking errors. FlyTracker could generate a background model and occasionally segment flies successfully, but detection remained unstable and the full pipeline failed during feature computation. These issues are particularly relevant for copulation latency and mating duration analysis: during copulation, the male and female remain physically coupled for a long period, which makes identity-based tracking difficult. For these timing metrics, it is more important to robustly detect the onset and offset of the mating state than to reconstruct detailed individual trajectories. DrosoMating was purpose-built to address these specific challenges. It operates reliably on lower-quality video streams, requires no complex pre-processing or manual ROI definition, and is optimized for high-throughput multi-chamber analysis. While it does not offer the same level of pose or kinematic detail as other tools, it provides a unique solution for laboratories seeking a simple, fast, and robust pipeline to quantify core reproductive timing metrics—copulation latency (CL), courtship index (CI), and mating duration (MD)—without the overhead of more complex systems (Table 1).

Although we directly tested only Ctrax and FlyTracker, this limitation may also affect workflows that depend on upstream tracking-derived features. For example, JAABA uses tracking-derived features to train behavior classifiers, and DANCE, a recent Drosophila aggression and courtship pipeline, uses JAABA-based classifiers and lists FlyTracker and JAABA as required software. Thus, our conclusion is not that these tools are generally unsuitable for Drosophila behavior analysis, but that DrosoMating provides a more practical workflow for low-quality, high-throughput videos focused specifically on mating timing.

While DrosoMating currently prioritizes mating timing metrics over discrete behavioral classification, its modular architecture provides a foundation for future integration with behavior classifiers such as JAABA. Laboratories requiring granular behavioral elements—such as wing extension or circling—would benefit from an extended pipeline that exports per-frame kinematic features for downstream classifier training. Validating this integration represents a promising future direction to broaden the tool’s utility beyond core reproductive timing assays.”

We have also added this clarification to the Table 1 legend to avoid overgeneralizing the limitations of Ctrax and FlyTracker (line 786-790):

"DrosoMating was designed to extract mating-related timing metrics from high-throughput videos without requiring continuous two-fly identity tracking. Ctrax and FlyTracker were tested on cropped single-chamber videos from the same recording setup. Their limitations described here refer specifically to these low-quality mating videos and should not be interpreted as general limitations of the tools."

(2) The coarse nature of the metrics captured by DrosoMating may limit the usefulness of this tool for many researchers. Consider integrating DrosoMating with one or more behavioral classifiers (e.g., JAABA) and validating its performance to increase its utility across a wider range of uses. Demonstrating the feasibility of this would substantially increase the tool's appeal to those who need access to more discrete behavioral elements.

We thank the reviewer for this constructive suggestion. We agree that DrosoMating is currently optimized for rapid, high-throughput quantification of core mating timing metrics (CL, CI, and MD) rather than discrete behavioral classification (e.g., wing extension, circling, or aggressive postures). This reflects a deliberate design trade-off: by prioritizing robust state detection over detailed pose tracking, DrosoMating achieves reliable performance on low-quality videos where conventional identity-based pipelines fail.

We appreciate the reviewer’s vision for expanding the tool’s utility. While DrosoMating does not presently generate the per-frame kinematic features (position, orientation, wing angles, etc.) required as input for JAABA classifiers, its modular architecture and underlying video-processing framework provide a foundation for future integration. In the revised Discussion, we have clarified that extending DrosoMating to export trajectory-derived features compatible with downstream classifiers such as JAABA represents a promising future direction—one that would broaden its applicability to laboratories requiring granular behavioral elements, without necessitating a complete overhaul of the existing pipeline.

We hope this clarification addresses the reviewer’s concern and accurately reflects both the current capabilities and future potential of the tool.

The revised manuscript text is as follows (line 614-620):

"While DrosoMating currently prioritizes mating timing metrics over discrete behavioral classification, its modular architecture provides a foundation for future integration with behavior classifiers such as JAABA. Laboratories requiring granular behavioral elements—such as wing extension or circling—would benefit from an extended pipeline that exports per-frame kinematic features for downstream classifier training. Validating this integration represents a promising future direction to broaden the tool’s utility beyond core reproductive timing assays."

(3) Please clarify how DrosoMating handles fly identification during mating? I would think mounted flies would be occluded, and if these frames are excluded from analysis, it would be expected to skew mating duration scores. It would be helpful if the authors could discuss these details.

Occluded frames are excluded from CI calculation because individual courtship actions cannot be reliably assigned during prolonged overlap. However, these frames are not discarded from mating-duration analysis. Instead, prolonged merged contours are used as evidence for copulation-state detection, and the MD timer continues until physical separation:

(1) When two flies are separate, the system detects two contours; when they overlap during mounting, it detects one merged contour. Our code processes both cases, so occluded frames are retained, not skipped.

(2) To distinguish a single fly from two overlapping flies, we analyze the shape of the merged contour. Two overlapping flies produce a characteristically different aspect ratio (elongated shape) compared to one fly. This geometric cue is fed into the state classifier to label the frame as "mating."

(3) Because these frames are classified as mating rather than excluded, the mating duration timer runs continuously through the occlusion period. Thus, mating duration is not artificially shortened.

The high agreement between DrosoMating and manual scoring for mating duration (within 10 s, no significant difference) confirms that this approach does not introduce bias.

We revised the manuscript as follow (line 191-193):

"Occluded frames are excluded from CI calculation but retained for mating-state detection via merged-contour analysis, ensuring continuous MD measurement through copulation."

(4) Please indicate the statistical tests used in Figures 3, 4, and S1. What methods were used to address multiple hypothesis testing?

We thank the reviewer for this important comment. For comparisons between two groups, two-sided Student’s t-tests were used. For comparisons among three or more groups, one-way ANOVA followed by post-hoc tests were applied. For two-factor experimental designs, two-way ANOVA was used. We have clearly stated these statistical tests in the Statistical Analysis section and have added this information to the figure legends for Figures 3, 4, and S1 in the revised manuscript.

The revised "Statistical Analysis" section is as follows (line 512-524):

"To ensure robust statistical analysis, each experimental group included at least 100 male flies (naïve, sexually experienced, or singly reared). Internal controls were incorporated into every experiment as recommended by Bretman et al. (2011) (Bretman et al., 2011). Normality of the mating duration data was confirmed using the Kolmogorov-Smirnov test (p>0.05). For group comparisons, two-sided Student’s t-tests were applied to calculate significance levels (****p<0.0001, ***p<0.001, **p<0.01, * p<0.05), Comparisons among three or more groups were performed using one-way ANOVA with Tukey’s HSD post-hoc tests. Two-factor experimental comparisons were performed using two-way ANOVA followed by Sidak’s post-hoc test. while estimation statistics (Claridge-Chang and Assam, 2016) were used to visualize effect sizes, mean differences, and precision, avoiding reliance solely on null hypothesis testing. All analyses, including data plotting, were performed using GraphPad Prism software."

(5) Line 43: "High resolution video tracking enables...".

We thank the reviewer for noting this imprecise expression. We have revised the statement to accurately describe that our method uses image-based video analysis to identify courtship and copulation events and quantify their temporal parameters. The description has been corrected in the revised manuscript line 44-46:

"Our image-based video analysis enables precise identification of courtship and copulation events, as well as quantification of their timing and duration under controlled conditions. "

(6) Line 51: remove quotation mark.

We thank the reviewer for the careful correction. The unnecessary quotation mark at Line 51 has been removed in the revised manuscript.

(7) Line 107: Chen et al, 2024 is listed twice in the references.

We thank the reviewer for the careful correction. The duplicate reference of Chen et al., 2024 has been removed from the reference list in the revised manuscript.

(8) Line 242: remove quotation mark.

We thank the reviewer for the careful correction. The unnecessary quotation mark at Line 242 has been removed in the revised manuscript.

**Reviewer #2 (Recommendations for the authors):**

(1) Please re-analyze the data in Figures 3 and 4 using ANOVA followed by appropriate post-hoc tests (e.g., Tukey's HSD). Specifically, use a One-way ANOVA for strain comparisons in Figure 3 and a Two-way ANOVA for the learning assays in Figure 4. The interaction term (Genotype $\times$ Training) is critical for demonstrating specific learning deficits. Update the "Statistical Analysis" section and figure legends accordingly.

We appreciate the reviewer’s recommendation. We have re-analyzed the data in Figures 3 and 4 using the suggested ANOVA approaches: one-way ANOVA with Tukey’s HSD for strain comparisons (Figure 3), and two-way ANOVA (including the Genotype × Training interaction term) with Sidak's post-hoc test for learning assays (Figure 4). The updated statistical methods are now described in the Statistical Analysis section and figure legends.

The revised "Statistical Analysis" section is as follows (line 512-524):

"To ensure robust statistical analysis, each experimental group included at least 100 male flies (naïve, sexually experienced, or singly reared). Internal controls were incorporated into every experiment as recommended by Bretman et al. (2011) (Bretman et al., 2011). Normality of the mating duration data was confirmed using the Kolmogorov-Smirnov test (p>0.05). For group comparisons, two-sided Student’s t-tests were applied to calculate significance levels (****p<0.0001, ***p<0.001, **p<0.01, * p<0.05), Comparisons among three or more groups were performed using one-way ANOVA with Tukey’s HSD post-hoc tests. Two-factor experimental comparisons were performed using two-way ANOVA followed by Sidak’s post-hoc test. while estimation statistics (Claridge-Chang and Assam, 2016) were used to visualize effect sizes, mean differences, and precision, avoiding reliance solely on null hypothesis testing. All analyses, including data plotting, were performed using GraphPad Prism software."

(2) To decouple motor defects from courtship deficits in $w^{1118}$ and $y^1$ mutants, please use your tracking data to calculate and present a "General Locomotion" metric (e.g., average velocity or total distance traveled in the absence of a female).

We thank the reviewer for this suggestion. We have now added Figure S4 showing basal locomotor velocity of single-housed males across all four strains. As expected, w^1118 and y^1 mutants move more slowly than wild-type controls.

These data reveal that both motor and sensory factors contribute to the observed courtship phenotypes. Reduced basal locomotion likely limits the males' ability to approach and follow females. However, this generalized hypoactivity is compounded by strain-specific sensory deficits: w^1118^ males suffer visual impairment that compromises female detection (Krstic et al., 2013), while y^1^ males display altered cuticular hydrocarbons that disrupt pheromonal communication (Drapeau et al., 2006). These sensory defects impair courtship initiation and female recognition independent of locomotor capacity. Thus, the reduced CI, CL, and MD in these mutants reflect the combined effects of slower movement and courtship-specific sensory impairments, rather than motor defects alone.

We have revised the manuscript to incorporate the velocity data and clarify this interpretation (line 219-226):

"Notably, reduced basal locomotor activity in w^1118^ and y^1^ mutants has been well documented in previous studies, independent of courtship behavior (Drapeau et al., 2006; Krstic et al., 2013). Consistent with these reports, our tracking data show that single-housed w^1118^ and y^1^ males exhibit lower average velocity than Canton-S and Oregon-R controls (Fig. S4A). These general locomotor differences are insufficient to fully explain the observed courtship and mating timing phenotypes, indicating that additional courtship‑related processes contribute to the observed behavioral differences."

(3) Please expand the discussion or provide a small comparative dataset contrasting DrosoMating with established tools like JAABA. Explain the specific advantages of your pipeline (e.g., cost, simplicity, focus on CL/MD) to justify its adoption over these alternatives.

We sincerely appreciate the reviewer’s insightful comments on benchmarking DrosoMating against existing tools for automated courtship behavior analysis. We fully agree that direct comparisons are essential to clarify the unique advantages and ideal use cases of DrosoMating. We have extensively revised the manuscript by adding comparative experiments, a new figure (Figure 5) and comparative table (Table 1), and expanded descriptions, as detailed below.

(1) Direct comparison with conventional tracking tools

We systematically tested Ctrax and FlyTracker on the same low-quality, high-throughput mating videos used for DrosoMating. The corresponding results are presented in Figure 5.

Ctrax showed severe segmentation instability, including over-segmentation, under-segmentation, and fragmented trajectories, and failed to stably detect two flies.

FlyTracker was able to generate background models and showed partially improved segmentation after threshold adjustment, but frequently crashed during feature computation and could not complete the end-to-end analysis pipeline.

These results confirm that tracking-based tools are vulnerable to low contrast, chamber artifacts, and prolonged male–female overlap, whereas DrosoMating bypasses pose tracking and directly detects mating states.

The revised manuscript text is as follows (line 265-342):

" DrosoMating is more compatible with low-quality mating videos than conventional tracking-based pipelines

To evaluate whether conventional fly-tracking pipelines could be used as alternative tools for extracting mating-duration metrics, we tested Ctrax and FlyTracker on representative low-quality single-chamber mating videos recorded under our standard high-throughput conditions (Fig. 5). Ctrax is designed to estimate the position and orientation of multiple walking flies while maintaining individual identities over time (Branson et al., 2009) (https://ctrax.sourceforge.net/), whereas FlyTracker aims to track fly pose, including position, orientation, body size, wing and leg positions, and to generate trajectory- and feature-based outputs for downstream behavior analysis (Eyjolfsdottir et al., 2014). Because these programs were not readily compatible with our full high-throughput behavioral recording setup, we first cropped the original videos and tested single-chamber videos containing one male and one female fly.

Using Ctrax, we observed that target detection was highly variable even within single-chamber videos. In representative frames, Ctrax could occasionally identify the two flies correctly (Fig. 5A). However, imperfect segmentation was frequently observed. In some frames, parts of the fly body were detected as additional targets, resulting in over-segmentation and an apparent increase in the number of detected flies (Fig. 5B, C). Conversely, when the male and female were close to each other or physically overlapped, the two animals were sometimes detected as a single target (Fig. 5D). These examples indicate that Ctrax detection was sensitive to the low contrast and overlapping fly bodies present in our mating videos. We then adjusted the Ctrax detection threshold to improve segmentation quality. Although threshold optimization improved detection in some frames, abnormal detections remained evident across randomly sampled frames (Fig. 5E). For example, some frames still showed incorrect target numbers, and a severe segmentation failure was observed in the lower-right example, where the detected objects did not correspond to two clearly separable flies. Thus, even after parameter optimization, Ctrax did not consistently maintain the expected two-target detection state in single-chamber mating videos.

The instability of Ctrax detection was also reflected in the trajectory output. In the first 500 frames, the generated trajectories were fragmented into multiple colored track segments rather than two continuous trajectories corresponding to the male and female (Fig. 5F). In addition, some trajectories extended outside the chamber boundary, indicating tracking errors and identity instability. Consistently, frame-by-frame quantification of detected target number showed that the detected object count did not remain stable at the expected value of two flies per chamber (Fig. 5G). Because Ctrax failed to maintain stable two-fly detection and continuous trajectories under these video conditions, its output could not be reliably used for downstream extraction of copulation latency or mating duration.

We next tested FlyTracker on the same type of cropped single-chamber mating videos. FlyTracker was developed to track multiple flies by estimating body position, orientation, size, wing and leg positions, and by maintaining fly identities across video frames; it also outputs per-frame features such as velocity, facing angle, and wing-angle-related measurements for downstream behavioral analysis (Eyjolfsdottir et al., 2014). In our videos, FlyTracker was able to generate a background model, indicating that the program could recognize the overall imaging field and chamber background (Fig. 5H). However, during calibration and segmentation, the default threshold setting produced inconsistent detection results (Fig. 5I). Although some frames were segmented relatively well under the default threshold (Fig. 5J), these successful examples were not representative of the overall tracking process, and the program frequently terminated with runtime errors during tracking or downstream feature computation. To improve detection stability, we lowered the segmentation threshold. Under this adjusted setting, FlyTracker produced more complete fly masks in representative frames (Fig. 5K), and the diagnostic output showed that two flies were detected in many sampled frames (Fig. 5L). Nevertheless, detection remained unstable in some sampled frames, including frames in which zero or one fly was detected despite the expected two flies per chamber (Fig. 5L). The full pipeline ultimately failed during feature computation, producing a runtime error before complete tracking and feature outputs could be generated (Fig. 5M). Therefore, even after threshold adjustment, FlyTracker could not provide a stable end-to-end workflow for extracting mating-duration metrics from these low-quality mating videos.

This failure mode is relevant because FlyTracker depends on stable segmentation, identity maintenance, and per-frame feature extraction. In our assay videos, low contrast, chamber-edge artifacts, and prolonged male–female overlap during copulation interfered with these requirements. As a result, FlyTracker could occasionally identify the flies in individual frames, but it did not reliably complete the full analysis pipeline required for downstream behavioral quantification. This limitation is especially important for workflows such as JAABA, which use manually labeled examples to train behavior classifiers but still depend on upstream tracking-derived features. Thus, for our low-quality high-throughput mating recordings, FlyTracker-based analysis was substantially less practical than DrosoMating, which directly outputs mating-related timing metrics without requiring continuous high-fidelity two-fly pose tracking."

(2) Clarification of ideal use cases

We revised the Discussion to emphasize that DrosoMating is not intended to replace general-purpose pose or tracking tools (e.g., FlyTracker, Ctrax) that provide fine-grained behavioral data.

Instead, it offers a specialized, robust, and high-throughput workflow for scenarios requiring efficient extraction of mating timing metrics from low-quality videos, where conventional pipelines often fail.

These revisions greatly improve the clarity and positioning of DrosoMating. We thank the reviewer for this valuable suggestion.

The revised manuscript text is as follows (line 578-620):

“Comparative Evaluation with Existing Courtship Analysis Tools

A major advantage of DrosoMating is its compatibility with low-quality, high-throughput mating videos. Conventional tracking-based tools such as Ctrax and FlyTracker are powerful for trajectory- and pose-based behavioral analysis, but they generally require stable object segmentation, identity maintenance, and reliable feature extraction across frames. Ctrax was designed to estimate the positions and orientations of multiple walking flies while maintaining their identities, whereas FlyTracker aims to track detailed fly pose and generate per-frame behavioural features. Under our recording conditions, these requirements were difficult to satisfy because the videos were low contrast and the male and female frequently overlapped during copulation.

In our tests, both tools showed limited compatibility with these videos. Even after cropping to single-chamber videos and adjusting detection parameters, Ctrax produced unstable target numbers, fragmented trajectories, and tracking errors. FlyTracker could generate a background model and occasionally segment flies successfully, but detection remained unstable and the full pipeline failed during feature computation. These issues are particularly relevant for copulation latency and mating duration analysis: during copulation, the male and female remain physically coupled for a long period, which makes identity-based tracking difficult. For these timing metrics, it is more important to robustly detect the onset and offset of the mating state than to reconstruct detailed individual trajectories. DrosoMating was purpose-built to address these specific challenges. It operates reliably on lower-quality video streams, requires no complex pre-processing or manual ROI definition, and is optimized for high-throughput multi-chamber analysis. While it does not offer the same level of pose or kinematic detail as other tools, it provides a unique solution for laboratories seeking a simple, fast, and robust pipeline to quantify core reproductive timing metrics—copulation latency (CL), courtship index (CI), and mating duration (MD)—without the overhead of more complex systems (Table 1).

Although we directly tested only Ctrax and FlyTracker, this limitation may also affect workflows that depend on upstream tracking-derived features. For example, JAABA uses tracking-derived features to train behavior classifiers, and DANCE, a recent Drosophila aggression and courtship pipeline, uses JAABA-based classifiers and lists FlyTracker and JAABA as required software. Thus, our conclusion is not that these tools are generally unsuitable for Drosophila behavior analysis, but that DrosoMating provides a more practical workflow for low-quality, high-throughput videos focused specifically on mating timing.

While DrosoMating currently prioritizes mating timing metrics over discrete behavioral classification, its modular architecture provides a foundation for future integration with behavior classifiers such as JAABA. Laboratories requiring granular behavioral elements—such as wing extension or circling—would benefit from an extended pipeline that exports per-frame kinematic features for downstream classifier training. Validating this integration represents a promising future direction to broaden the tool’s utility beyond core reproductive timing assays.”

We have added this clarification to the Table 1 legend to avoid overgeneralizing the limitations of Ctrax and FlyTracker (line 786-790):

"DrosoMating was designed to extract mating-related timing metrics from high-throughput videos without requiring continuous two-fly identity tracking. Ctrax and FlyTracker were tested on cropped single-chamber videos from the same recording setup. Their limitations described here refer specifically to these low-quality mating videos and should not be interpreted as general limitations of the tools."

(4) Complement the aggregate bar plots with behavioral ethograms or raster plots for representative individual flies. Color-code these plots for specific states (resting, following, courting, copulating) to visually demonstrate the system's temporal resolution and detection accuracy.

We thank the reviewer for this valuable suggestion. To address this comment, we have added a new supplementary figure (Figure S3) that presents behavioral ethograms for individual flies, directly complementing the aggregate bar plots in the main text. The figure displays the full temporal progression of courtship and copulation behaviors for all wells that exhibited successful mating. As requested, behaviors are color-coded (orange: courting, red: copulating) to clearly delineate different states. These ethograms visually demonstrate the system’s ability to resolve behavioral transitions with high temporal precision, confirming the accuracy of our automated detection of courtship initiation, duration, and copulation events. This addition provides critical individual-level validation that supports the aggregate statistical results presented in the main text.

(5) Standardize the use of estimation statistics (DBMs). If used in Figure 3, they should also be applied to Figure 4, with appropriate statistical comparisons between groups.

We thank the reviewer for this suggestion. We have standardized the statistical reporting format across all figure legends, with each legend explicitly stating the test used, the post hoc method (where applicable), and significance thresholds.

Our approach is as follows:

Fig. 2 and Fig.3D-I (two-group comparison, manual vs. automated scoring): DBM + Student's t-test.

Fig. 3A-C and Fig. S1B-C, E-F (multi-group comparison, 4 strains or 2 rearing conditions): One-way ANOVA + Tukey's HSD.

Fig. 4B-D and F (two-factor design, genotype × training): Two-way ANOVA + Sidak's post hoc.

We have also added a sentence to the Methods clarifying that estimation statistics (DBM) are used for single two-group contrasts, while ANOVA-based approaches are used for multi-group or multi-factor designs. The statistical methods are now consistently documented in both the Methods section and the corresponding figure legends. We hope this clarification addresses the reviewer's concern.

The revised "Statistical Analysis" section is as follows (line 512-524):

"To ensure robust statistical analysis, each experimental group included at least 100 male flies (naïve, sexually experienced, or singly reared). Internal controls were incorporated into every experiment as recommended by Bretman et al. (2011) (Bretman et al., 2011). Normality of the mating duration data was confirmed using the Kolmogorov-Smirnov test (p>0.05). For group comparisons, two-sided Student’s t-tests were applied to calculate significance levels (****p<0.0001, ***p<0.001, **p<0.01, * p<0.05), Comparisons among three or more groups were performed using one-way ANOVA with Tukey’s HSD post-hoc tests. Two-factor experimental comparisons were performed using two-way ANOVA followed by Sidak’s post-hoc test. while estimation statistics (Claridge-Chang and Assam, 2016) were used to visualize effect sizes, mean differences, and precision, avoiding reliance solely on null hypothesis testing. All analyses, including data plotting, were performed using GraphPad Prism software."

(6) Fix inconsistent labeling (e.g., "MB-247" vs. "MB247") and redundant axis labels.

Thank you for pointing this out. We have now standardized all labels to "MB247-GAL4" throughout the text and figures.

We have also removed redundant axis labels from multi-panel figures. And redundant DBM and metric labels have been streamlined in Figures 2 and 4. All statistical tests and metric definitions are now fully described in the corresponding figure legends rather than being repeated on each sub-panel.

(7) Significantly increase the size of data panels to make individual data points and error bars legible.

Thank you for this suggestion. We have standardized the figure formatting and simplified the panels by removing redundant axis labels and consolidating descriptive details into the figure legends. We believe the current panel sizes, combined with these clarifications, provide sufficient legibility for both data points and error bars in the final high-resolution PDF.

Minor Corrections:

(1) Abstract: Rephrase "lack of certain timing-related behavioral repertoires" (Line 108) to "lack of precise quantification for temporal parameters of post-copulatory behavior."

Thank you for the suggested rephrasing. We have updated the sentence accordingly.

(2) Define the physical parameter "s" (e.g., is it a pixel threshold?) to ensure reproducibility.

Thank you for this important suggestion. We have now explicitly defined the parameter s in the Methods section. Briefly, s is the grayscale intensity threshold (0–255, 8-bit) used for binary segmentation of flies from the background. It is automatically calculated as the maximum grayscale value of three user-selected reference flies plus an offset of 28, and can be manually adjusted to accommodate varying illumination conditions.

The revised manuscript text is as follows (line 499-503):

“Note on the s value: The parameter s represents the grayscale intensity threshold (range: 0–255 for 8-bit images) used for binary segmentation of flies from the background. It is automatically calculated as the maximum grayscale value at the three reference fly positions plus an offset of 28, and can be manually adjusted to accommodate varying illumination conditions.”

(3) Figure 1 Legend: Clarify the "clockwise selection" of pillars and their relation to well numbering.

Thank you for this suggestion. We have revised the Figure 1 legend to clarify that:

(1) The four pillars are selected in clockwise order starting from the top-left corner to define the chamber corners for perspective transformation (homography), which corrects for camera angle and standardizes the field of view.

(2) Well numbering is independent of pillar selection order. After automated perspective correction, wells are numbered sequentially in a left-to-right, top-to-bottom order (1–36) based on the standardized chamber layout.

The updated Figure 1 legend now reads:

"Columns (pillars) should be selected in clockwise order starting from the top-left corner to define the chamber boundaries for perspective transformation (Fig. 1A, lower). Well numbering (1–36) follows a left-to-right, top-to-bottom sequence after perspective correction and is independent of pillar selection order."

(4) Add the citation for Eastwood and Burnet (1977) regarding Courtship Latency.

Thank you for pointing this out. We have added the citation Eastwood and Burnet (1977) to the Introduction where Courtship Latency is first defined

(5) Select one term ("Mating Duration" or "Copulation Duration") and use it consistently throughout the text and figures.

Thank you for this suggestion. We have now standardized the terminology throughout the manuscript and figures. "Mating Duration" (MD) is used consistently in all instances where "Copulation Duration" previously appeared. The abbreviation MD has been retained for consistency with existing figure labels

References

Branson, K., Robie, A.A., Bender, J., Perona, P., & Dickinson, M.H. (2009). High-throughput ethomics in large groups of Drosophila. Nature Methods, 6(6), 451–458.

Claridge-Chang, A., & Assam, P.N. (2016). Estimation statistics should replace significance testing. Nature Methods, 13(2), 108–109.

Drapeau, M.D., Cyran, S.A., Viering, M.M., Geyer, P.K., & Long, A.D. (2006). A cis-regulatory sequence within the yellow locus of *Drosophila melanogaster* required for normal male mating success. Genetics, 172(2), 1009–1030.

Eastwood, L., & Burnet, B. (1977). Courtship latency in male *Drosophila melanogaster*. Behavior Genetics, 7(3), 359–372.

Eyjolfsdottir, E., Branson, S., Burgos-Artizzu, X.P., Hoopfer, E.D., Schor, J., Anderson, D.J., & Perona, P. (2014). Detecting social actions of fruit flies. Lecture Notes in Computer Science, 8692, 772–787.

Gil-Martí, B., Barredo, C.G., Pina-Flores, S., Poza-Rodriguez, A., Treves, G., Rodriguez-Navas, C., Camacho, L., Pérez-Serna, A., Jimenez, I., Brazales, L., Fernandez, J., & Martin, F.A. (2023). A simplified courtship conditioning protocol to test learning and memory in Drosophila. STAR Protocols, 4(1), 101572.

Greenspan, R.J., & Ferveur, J.F. (2000). Courtship in Drosophila. Annual Review of Genetics, 34, 205–232.

Hall, J.C. (1994). The mating of a fly. Science, 264(5163), 1702–1714.

Kabra, M., Robie, A.A., Rivera-Alba, M., Branson, S., & Branson, K. (2013). JAABA: interactive machine learning for automatic annotation of animal behavior. Nature Methods, 10(1), 64–67.

Kitamoto, T. (2001). Conditional modification of behavior in Drosophila by targeted expression of a temperature-sensitive shibire allele in defined neurons. Journal of Neurobiology, 47(2), 81–92.

Krstic, D., Boll, W., & Noll, M. (2013). Influence of the White locus on the courtship behavior of Drosophila males. PLoS ONE, 8(9), e77904.

Levin, L.R., Han, P.L., Hwang, P.M., Feinstein, P.G., Davis, R.L., & Reed, R.R. (1992). The Drosophila learning and memory gene rutabaga encodes a Ca2+/calmodulin-responsive adenylyl cyclase. Cell, 68(3), 479–489.

Pavlou, H.J., & Goodwin, S.F. (2013). Courtship behavior in *Drosophila melanogaster*: towards a ‘courtship connectome’. Current Opinion in Neurobiology, 23(1), 76–83.

Yapici, N., Kim, Y.J., Ribeiro, C., & Dickson, B.J. (2008). A receptor that mediates the post-mating switch in Drosophila reproductive behaviour. Nature, 451(7176), 33–37.
